# Fatigue in Systemic Lupus Erythematosus: An Update on Its Impact, Determinants and Therapeutic Management

**DOI:** 10.3390/jcm10173996

**Published:** 2021-09-03

**Authors:** Lou Kawka, Aurélien Schlencker, Philippe Mertz, Thierry Martin, Laurent Arnaud

**Affiliations:** 1 Department of Rheumatology, INSERM UMR-S1109, Hôpitaux Universitaires de Strasbourg, 67000 Strasbourg, France; lou.kawka@chru-strasbourg.fr (L.K.); aurelien.schlencker@chru-strasbourg.fr (A.S.); Philippe.mertz@chru-strasbourg.fr (P.M.); 2 Centre National de Référence des Maladies Auto-Immunes Systémiques Rares Est Sud-Ouest (RESO), 67000 Strasbourg, France; Thierry.martin@chru-strasbourg.fr; 3 Department of Clinical Immunology, Hôpitaux Universitaires de Strasbourg, 67000 Strasbourg, France

**Keywords:** systemic lupus erythematosus, fatigue, quality of life

## Abstract

Fatigue is a complex and multifactorial phenomenon which is often neglected by clinicians. The aim of this review was to analyze the impact, determinants and management of fatigue in patients with Systemic Lupus Erythematosus (SLE). Fatigue is one of the most prevalent symptoms in SLE, reported by 67% to 90% of patients. It is also described as the most bothersome symptom, considering that it may impair key aspects of health-related quality of life, while also leading to employment disability. It is a multifactorial phenomenon involving psychological factors, pain, lifestyle factors such as reduced physical activity, whereas the contribution of disease activity remains controversial. The management of fatigue in patients with SLE should rely upon a person-centered approach, with targeted interventions. Some pharmacological treatments used to control disease activity have demonstrated beneficial effects upon fatigue and non-pharmacological therapies such as psychological interventions, pain reduction and lifestyle changes, and each of these should be incorporated into fatigue management in SLE.

## 1. Introduction

Fatigue is a universal symptom experienced by nearly everyone in the general population. However, we lack a consensual definition of fatigue. Fatigue can be described as a subjective unpleasant sensation of exhaustion with physical and mental components, which interferes with individuals’ ability to function at their normal capacity. Fatigue impairs quality of life, and may lead to irritability, inability to concentrate, and poor motivation [1,2]. In chronic conditions such as Systemic Lupus Erythematosus (SLE), but also in other autoimmune diseases such as Sjögren’s syndrome or systemic sclerosis, the experience of fatigue seems to differ from ‘everyday tiredness’, as being more frequent, unpredictable and typically unresolved by rest [3]. This symptom remains a complex, multidimensional and poorly understood concept, often neglected by clinicians who prefer to focus on objective manifestations. The aim of this review was to report upon the impact, determinants and management of fatigue in patients with SLE.

## 2. The Most Frequent and Disabling Symptom

Fatigue is recognized as one of the most prevalent symptoms in SLE, reported by 67% to 90% of patients, depending on the series [4]. In a 2020 survey analyzing the burden of SLE from the patients’ perspective in European countries [4], fatigue was described as the most common symptom (affecting 85.3% of the 4375 respondents) and the most bothersome symptom, which is consistent with previous studies. Fatigue is reported as severe in intensity in more than a third of SLE patients [4].

Fatigue may impair several key aspects of the patient’s quality of life, with repercussions on both physical and mental health. Indeed, SLE patients report that fatigue has a negative impact on emotions, cognition, work, activities of daily living, leisure activities, social activities and family activities. They describe physical impairment, with walking and exercising difficulties. Emotional consequences of fatigue, such as frustrations and stress due to being unable to accomplish tasks, sadness or loss of motivation, are also common [5,6,7,8,9]. Fatigue in SLE has a significantly negative impact on work ability and work productivity, as it can lead to limitations in workplace activities by affecting endurance, mobility, concentration, or interactions with employees and coworkers. Fatigue in SLE is also associated with a higher risk of absenteeism and unemployment [10]. Altogether, fatigue is an important determinant in the perception of SLE impact upon patients’ daily living, even for those in remission.

## 3. A Multifactorial Manifestation

**Figure 1:**
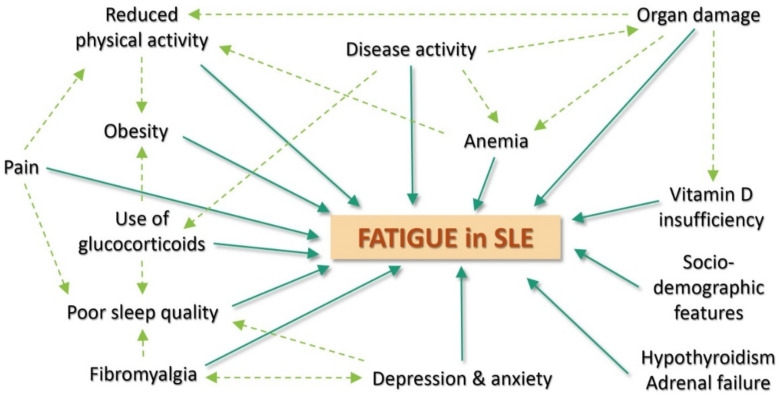
Main determinants of fatigue in Systemic Lupus Erythematosus.

Altogether, fatigue is a highly multifactorial manifestation (Figure 1), caused by a complex interplay between disease itself, psychosocial, behavioral and personal variables. A recent study from our group described 3 main clusters of fatigue in SLE patients: (1) the most frequent profile (67.5% of the patients) was represented by patients with moderate fatigue, low disease activity and low anxiety and depression; (2) a quarter of the patients had very high fatigue, high depression and anxiety but low disease activity; and (3) less than 10% of the patients had high levels of fatigue, with high disease activity, low anxiety and no depression [11]. This suggests that the mental health status is an important predictor of fatigue in SLE, that disease activity plays a weaker role in SLE fatigue, and that, most of the time, other factors contribute to fatigue in SLE.

### 3.1. Lupus-Related Determinants

The association between fatigue and disease activity in SLE has been widely studied and debated for a long time, with controversial results [11,12,13,14,15,16,17,18,19]. Type I interferons, which are key cytokines in SLE, are associated with fatigue and may provide a clue towards a pathogenic explanation for fatigue in SLE. Disease activity seems to play a role in the genesis of fatigue but it cannot fully explain fatigue by itself. Indeed, a study based on an inception cohort of adult patients with SLE found that fatigue and disease activity followed distinct trajectories over 10 years [20]. Additionally, in our recent FATILUP studies [21], the association between fatigue and disease activity was significant, but weak (OR: 1.05 (95% CI: 1.00–1.12) per 1 point increase in SELENA-SLEDAI score). Therefore, it is likely that disease activity has a complex and potentially indirect contribution to fatigue, such as by influencing other major determinants of fatigue, for example pain and psychological factors. Some specific organ involvements such as neurological impairment and painful manifestations such as arthritis or oral ulcers have been found to be associated with fatigue in some studies [14,21,22]. Pain has been reported to have a specific role in SLE fatigue, and chronic pain treatment is essential to the management of fatigue in SLE [12,14,17,23]. Organ damage, especially renal or cardiac failure, can also be important causes of fatigue in SLE patients [24,25,26,27]. Furthermore, the use of glucocorticoid has been shown to be independently associated with fatigue in SLE [21].

### 3.2. Psychological Determinants

Mental health status, emotional and functional wellbeing have shown to be major determinants of fatigue in SLE patients. Depression and anxiety appear to be among the strongest predictors of fatigue in SLE patients [11,12,13,21,23,28,29]. There is a clear association between fatigue and depression in general, and scales assessing depression often include fatigue-related items. Depression affects both physical and mental dimensions of fatigue in SLE. Additionally, depression is frequent in SLE patients (between 17 and 75% of patients), and some authors have mentioned that SLE contributes to depression through its neurological involvement, an autoimmune effect, and the emotional consequences of pain and disability [30,31,32]. Stress, which is a subjective negative perception of life events, which may be influenced by sociological and psychological factors and SLE burden, seems to mediate the relationship between depression and fatigue over time in SLE patients. Decline in stress has been associated with a meaningful improvement in fatigue in SLE [31]. Sleep disorders have also been shown to be common and significant predictors of fatigue, occurring in more than half of SLE patients [28,29,33,34]. SLE may contribute to sleep disorders because of pain and inflammation, and steroid use has been associated with sleep disorders [35]. In addition, helplessness (a state in which a person remains passive in negative situations), coping disability (difficulties in facing problems in an adequate manner) and abnormal illness-related behavior have been associated, although not independently, with fatigue in SLE in some series [14,15,19]. The role of psychological determinants is therefore major in SLE fatigue. Consequently, it is crucial to suggest a thorough psychological assessment of SLE patients reporting severe fatigue, especially for those with no or low disease activity, since mood disorders are frequent in patients with SLE [4,21,30,31,32] and multifactorial.

### 3.3. Comorbidities

Fibromyalgia is a major predictor of fatigue in SLE [14,18,36]. In a study conducted by Touma et al., trajectories with higher fatigue scores were associated with a higher prevalence of fibromyalgia [20]. Fibromyalgia is common in SLE patients (from 6.2% to 30% of patients) but may be underdiagnosed by physicians [21,37]. Consequently, the role of fibromyalgia should be considered in SLE patients who complain about fatigue and widespread pain. Other frequent comorbidities such as anemia, hypothyroidism, or adrenal failure are risk factors in fatigue. Vitamin D insufficiency was associated with fatigue in SLE in some but not all studies [38,39]. SLE patients have a high risk of vitamin D deficiency because of photoprotection as well as in case of renal failure.

### 3.4. Behavioral and Socio-Demographic Features

Reduced levels of physical activity and aerobic capacity significantly increase fatigue in SLE. SLE patients have many actual and perceived barriers to exercise. It has been shown that, compared to sedentary controls, SLE patients have reduced levels of aerobic fitness, reduced exercise capacity and reduced muscle strength, which further leads to a reduced ability to perform physical activity. Furthermore, SLE patients are limited by arthralgia, anemia, and other SLE organ involvements. For all of those reasons, SLE patients often have limited physical activity and assume a sedentary lifestyle [40,41,42]. Obesity and smoking are other potential behavioral determinants of fatigue in this population [14,43]. The role of sociodemographic features is contradictory, but some studies found higher levels of fatigue in SLE patients with low annual income, low education level, or difficulty in accessing health care [14,15]. In some studies, a low level of perceived social support was also associated with fatigue [12].

## 4. Interventions to Improve Fatigue

Recently, an increasing number of interventional studies focused on fatigue in SLE, and some pharmacologic and non-pharmacologic therapies have demonstrated beneficial effects on fatigue. Improving disease activity is associated with significant reduction in fatigue in randomized controlled trials of belimumab, blisibimod, and hydroxychloroquine [44,45,46]. This effect is likely to be observed with any treatment improving disease activity in SLE, although this has not been formally proven. N-acetyl-cysteine has also been shown to improve fatigue in SLE. A double-blind, placebo controlled, randomized trial found that a 2.4 g/day dose of N-acetyl-cysteine is effective for reducing fatigue and improving disease activity, and is safe and well-tolerated [47]. Vitamin D supplementation also seems to have positive effects on fatigue in SLE patients. An observational study found significantly lower fatigue scores after vitamin D supplementation in 80 SLE patients, and a randomized double-blind placebo-controlled trial showed a decrease in fatigue in juvenile-onset SLE patient receiving vitamin D supplementation [48,49]. Physical activities such as supervised training, home training, and appropriately prescribed graded aerobic exercise, have been associated with favorable improvements in patient-reported fatigue in different studies. Importantly, exercise was reported to be safe and well tolerated, with rare adverse effects, and no reported deleterious effects on disease activity or inflammation [50,51,52]. Physical activity should therefore be generally recommended for the management of fatigue in SLE patients, especially since it also leads to less pain interference, better physical function, cardiovascular risk reduction, and even positive impact on anxiety and depression. A trial conducted by Davies et al. indicates that a low glycemic index diet and a low-calorie diet were both associated with reduction in fatigue in SLE, indicating the role of weight loss in the improvement in fatigue [53].

Different psychosocial interventions have been associated with significant improvement in fatigue in SLE: cognitive behavioral therapy, psychoeducation, psychotherapy, relaxation and self-management. Those interventions focus on coping ability improvement, cognitive restructuring and perceived social supports [52,54,55,56]. Even if the effect in reducing fatigue has been shown to be weak in most of these studies, such interventions can decrease psychological distress and pain and therefore might be integrated into the general management of SLE patients. Some interventions targeting pain have also shown their ability to improve fatigue in patients with SLE. A randomized trial found a significant decrease in fatigue in SLE patients receiving transcutaneous auricular vagus nerve stimulation [57]. Additionally, a randomized controlled trial indicates benefits of acupuncture in reducing fatigue in patients with SLE [58].

## 5. The Need for a Personalized Management

**Figure 2:**
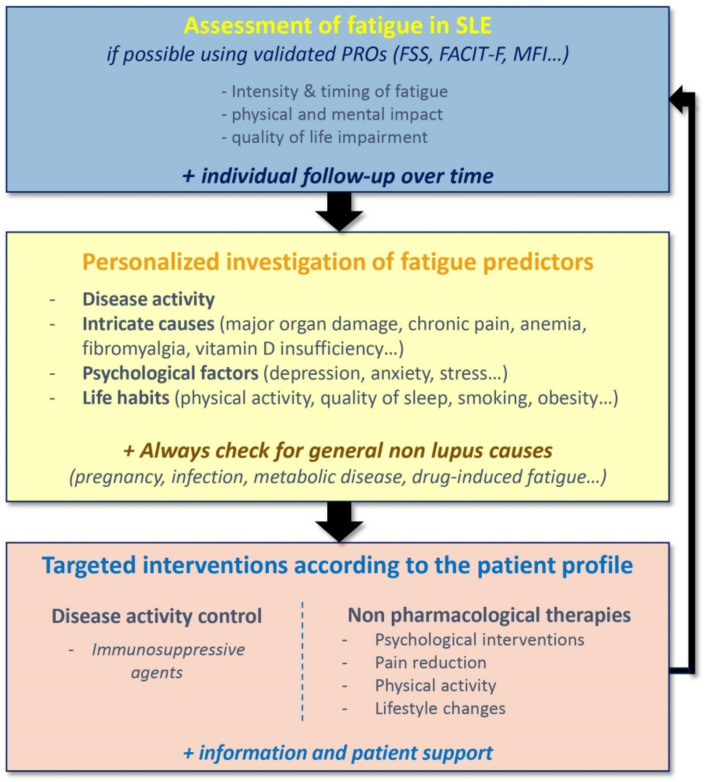
Personalized strategy for the assessment of fatigue in Systemic Lupus Erythematosus. FSS: Fatigue Severity Scale; FACIT-F: Functional Assessment of Chronic Illness Therapy—Fatigue; MFI: Multidimensional Fatigue Inventory.

At this time, there is no validated recommendation for the management of fatigue in SLE. Since fatigue may be influenced by a variety of factors and because of the diverse profiles of fatigue in SLE, the management of fatigue should rely upon an individualized person-centered approach (Figure 2). Women with SLE have reported the need for fatigue acknowledgement by clinicians, as well as conversations about fatigue, with information about coping strategies [59]. Fatigue management in SLE would start with an assessment of the intensity and the characteristics of fatigue using validated scales, enabling an individual follow-up of fatigue over time. In recent years, there has been an increasing interest in using Patient Reported Outcomes (PROs), because they place the patients at the center of their health management and help to establish a trusting physician-patient relationship. The most commonly PROs used to evaluate fatigue in SLE are the Fatigue Severity Scale (FSS), the FACIT-fatigue score, which we use in clinical practice, the Fatigue-VAS, which are unidimensional scales measuring fatigue intensity, and the Multi-dimensional Fatigue Inventory (MFI), which analyze general fatigue, physical and mental components of fatigue as well as the reduction in activities and motivation [60]. A personalized investigation of fatigue predictors is needed, with evaluation of disease activity, search for intricate causes (major organ damage, chronic pain, anemia…) and psychosocial factors, assessment of life habits (physical activity, quality of sleep, smoking, obesity…). Common medical causes of fatigue, such as pregnancy, infections, metabolic diseases or drug-induced fatigue must not be forgotten. Finally, optimal management of fatigue for patients with SLE should be based on providing targeted interventions, according to the patient profile [61].

## 6. Conclusions

According to patients, fatigue is the most common and disabling symptom in SLE, and this may impair patients’ physical and mental health and reduce patients’ quality of life by impacting upon their emotions, work, and daily life activities. Fatigue must therefore be adequately assessed and managed in SLE. It is a complex and multifactorial phenomenon, possessing many patterns. Psychological factors seem to be the most important fatigue predictors in SLE patients. Pain and fibromyalgia are also major fatigue determinants, along with lifestyle, especially reduced physical activity. Disease activity seems to have a complex contribution to fatigue, and its role remains controversial. Consequently, the management of fatigue in patients with SLE should rely upon a person-centered approach, with a personalized assessment, and targeted interventions. Some pharmacological treatments used to control disease activity, such as Belimumab, have demonstrated beneficial effects on fatigue. Non-pharmacological therapies, such as psychological interventions, pain reduction and lifestyle changes should be integrated into fatigue management in SLE. In recent years, the scientific community seems to have increased their understanding of the importance of fatigue management in SLE, and we can hope for a better understanding and treatment of fatigue in patients with SLE in the future.
